# Dyadic coping and related factors among couples with colorectal cancer: A latent profile analysis

**DOI:** 10.1016/j.apjon.2024.100571

**Published:** 2024-08-12

**Authors:** Tingting Wei, Qiao Feng, Tingting A, Shaohua Hu, Ping Ni, Dongmei Zhuang, Shihui Yu

**Affiliations:** a School of Nursing, Anhui Medical University, Hefei, China; b The First Affiliated Hospital of Anhui Medical University, Hefei, China; c Suzhou Hospital of Anhui Medical University, Suzhou, China

**Keywords:** Colorectal cancer, Spouse, Latent profile analysis, Dyadic coping, Couple illness communication, Fear of cancer recurrence

## Abstract

**Objective:**

This study aimed to identify latent subgroups of dyadic coping (DC) among colorectal cancer (CRC) patients and their spousal caregivers, and to explore the factors associated with these subgroups.

**Methods:**

We conducted a cross-sectional study involving 268 pairs of CRC patients and their spousal caregivers. Participants completed the General Information Questionnaire, the Dyadic Coping Inventory, the Cancer-Related Communication Problems Scale, and the Fear of Progress Questionnaire-Short Form. Latent profile analysis (LPA) of DC among CRC couples was performed using Mplus 8.3. We compared couple illness communication, fear of cancer recurrence (FCR), and demographic characteristics between the identified subgroups and conducted ordinal logistic regression analysis to examine factors associated with these subgroups.

**Results:**

The 268 pairs of CRC patients and their spousal caregivers were classified into four subgroups based on their coping levels: low-DC group (12.3%), low common-DC group (7.1%), moderate-DC group (52.6%), and high-DC group (28.0%). Disease stage, couple illness communication, and spouse's FCR were significantly associated with the four subgroups.

**Conclusions:**

There is considerable variability in DC levels among CRC patients and their spousal caregivers. Patients with advanced disease stages, inadequate communication between spouses, and severe RCR exhibit lower levels of DC. These findings provide a theoretical basis for nursing personnel to develop personalized intervention strategies tailored to the characteristics of these subgroups.

## Introduction

Colorectal cancer (CRC), including colon cancer and rectal cancer, is the third most common cancer globally and the second leading cause of cancer-related death.^1^ With the improvement of living standards and changes in people's lifestyles and dietary structures, the incidence and mortality of CRC are on the rise.^2^ According to the “Global Cancer Statistics 2020” report, in 2020, there were approximately 1.93 million new cases of CRC worldwide, with 0.94 million deaths, accounting for about one-tenth of cancer cases and deaths.^3^ In recent years, the incidence and mortality of CRC in China have also been increasing, and the burden of the disease will continue to rise. It is estimated that by 2025, the number of new cases of CRC in China will reach 0.64 million, with 0.22 million deaths.^4^

Research shows that the diagnosis and treatment of tumors have a dual-level impact on both spouses.^5^ Long-term wearing of stomas after CRC surgery and severe reactions to radiotherapy and chemotherapy impose a significant burden on patients.^6^ Spouses, as primary caregivers, are more likely to make sacrifices in caring for patients and bear serious physical and mental pressure in the treatment and recovery of the disease.^7^^,^^8^ The diagnosis of CRC as a stressor stimulates patients and spousal caregivers to produce certain coping behaviors. They perceive cancer as “our” disease and develop coping strategies as a whole.^9^ Dyadic coping (DC) refers to the common responses and decisions made by both spouses when facing stress.^10^ Mutual supportive DC can not only alleviate the negative effects of cancer but also promote the physical and mental health of both spouses and improve their quality of life.^11^

With the continuous advancements in medical technology and early cancer screening, the five-year survival rate for CRC patients can reach 65%.^12^ However, studies have shown that even after achieving surgical curative resection, up to 50% of CRC patients are at risk of metastasis and recurrence.^13^ Cancer recurrence and metastasis remain the biggest challenges faced by patients and their spousal caregivers. Fear of cancer recurrence (FCR) is one of the most common issues affecting the quality of life of patients and is also one of the most severe psychological problems faced by CRC patients and their spousal caregivers.^14^^,^^15^ Perndorfer found through diary studies that DC between breast cancer patients and their spousal caregivers is negatively correlated with both parties' FCR recurrence; the more severe the FCR, the lower the perceived spousal support.^16^

Communication is one of the key factors in determining whether couples can successfully cope with cancer together.^17^ The intimacy relationship model for couples facing cancer emphasizes that the patient-spouse pair, as an interdependent emotional system, can alleviate the cancer-related distress experienced by both parties through their emotional, cognitive, and behavioral characteristics.^18^ Couple illness communication is an important process of DC that can help couples adjust psychologically after the diagnosis and treatment of the disease.^19^ Wertheim used the Actor-Partner Interdependence Model to study the impact of couple illness communication on DC. The results showed that the better the communication between spouses, the higher the level of DC.^20^

The above research results indicate that FCR and couple illness communication are influencing factors of DC.^16^^,^^21^ However, these studies mostly focus on exploring factors related to DC in breast cancer patient couples and do not include CRC patients and their spousal caregivers.^22^ The number of CRC patients is large, with a mortality rate even higher than that of breast cancer patients.^1^ Patients and their spousal caregivers experience various issues during the disease process,^23^ such as common postoperative complications like sexual dysfunction and the significant burden of stoma care for patients with colostomies, which greatly affects spousal support and coping.^24^ Therefore, it is necessary to study the DC of CRC patients and their spousal caregivers and the related factors to improve the coping level between couples.

Currently, both domestic and international research on DC of CRC patients and their spousal caregivers is “variable-centered,” relying solely on total scores from scales to judge overall levels, neglecting the heterogeneity among individuals at different levels. Latent profile analysis (LPA), based on the concept of “person-centered” research, considers couples as a whole in coping with the disease.^25^ By considering the interaction between spouses, LPA categorizes couples into different performance subgroups. This method classifies the population probabilistically, identifying individuals who repeatedly exhibit the same pattern of continuous observable variables, thus dividing individuals within heterogeneous populations into smaller, more homogeneous groups.^26^ This approach can better understand the characteristics of latent subgroups and assess the proportional representation of different subgroups within the overall population, thereby capturing characteristics and inequalities among different categories of people that “variable-centered” approaches cannot observe.^27^ This provides a theoretical basis for precise interventions targeting DC of CRC patients and their spousal caregivers.

Therefore, the purpose of this study was to identify the potential subgroups of DC among CRC patients and their spousal caregivers by using LPA, determine the influencing factors of different subgroups to clarify subgroup characteristics, and provide a basis for developing nursing interventions to improve DC among CRC patients and their spousal caregivers.

## Methods

### Study design and participants

This study is a cross-sectional study, using convenience sampling. Between August 2023 and April 2024, 268 pairs of patients with CRC and their spouses were recruited from the department of gastroenterology and oncology of two tertiary Grade A hospitals in Anhui Province. The inclusion criteria were as follows: (1) Patients and spousal caregivers were married couples (≥ 18 years old) with one partner diagnosed with CRC, (2) patients were currently receiving treatment, (3) spouses assumed the major caregiving role, (4) patients and spouses were equipped with normal understanding and communication skills, and (5) patients and spouses agreed to participate in the study. The exclusion criteria were as follows: (1) One or both spouses had a history of cognitive impairment or mental illness, (2) one or both spouses had severe illness or physical disabilities, and (3) one or both spouses were unaware of the patient's condition. This study was not registered.

According to the Kendall sample size estimation method,^28^ the sample size should be at least 5–10 times the number of independent variables. This study included 21 independent variables, and considering a 10% inefficiency rate, 116–231 pairs of CRC patients couples were required. A total of 268 CRC patient couples were finally included in this study.

### Measures

#### General information questionnaire

Self-reported sociodemographic variables from patients and spousal caregivers were collected and included gender, age, religion, employment status, education, medical insurance type, place of residence, monthly income per capita, duration of marriage, number of children, and number of chronic diseases in the spouse. Medical characteristics were collected from medical records and included diagnosis duration, cancer stage, type of stoma, and type of chemotherapy.

#### Dyadic Coping Inventory

The Dyadic Coping Inventory (DCI) was developed by Bodenmann and was translated into Chinese by Xu in 2016 for the assessment of DC levels of patients and spouses.^29^^,^^30^ The scale consists of 35 items across five dimensions, including stress communication (8 items), supportive coping (10 items), delegated coping (4 items), negative coping (8 items), and common coping (5 items). It adopts a Likert 5-point rating scale, with the 8 items of negative coping scored in reverse, where higher scores indicate more supportive behaviors. The total score ranges from 35 to 175. Scores below 111 indicate a low level of DC, 111–145 indicate a moderate level, and scores above 145 indicate a high level of DC. The original scale had a Cronbach's α of 0.80, while in this study, the Cronbach's α of patient and spouse scales were 0.829 and 0.824.

#### Cancer-Related Communication Problems Scale

The Cancer-Related Communication Problems Scale (CRCP) was developed by Kornblith and was translated into Chinese by Li in 2016 to assess illness communication of cancer patients and spouses.^31^^,^^32^ The scale consists of 15 items, with the patient scale including emotional support (4 items), addressing specific concerns (4 items), self-protection (4 items), and protective buffering (3 items) across 4 dimensions, and the spouse scale including emotional support (4 items), addressing specific concerns (3 items), protective buffering (3 items), closed communication (3 items), and avoidance communication (2 items) across 5 dimensions. It adopts a Likert 3-point rating scale, where higher total scores indicate more severe cancer-related communication issues between spouses. The Cronbach's α of patient and spouse scales were 0.87, 0.81 in the original research and 0.920, 0.916 in this study.

#### Fear of Progress Questionnaire-Short Form

The Fear of Progress Questionnaire-Short Form (FoP-Q-SF) was developed by Mehnert and was translated into Chinese by Wu to assess the FCR in patients and their spouses.^33^^,^^34^ The scale consists of 12 items, with the patient scale divided into two dimensions: fear of physical health (6 items) and fear of social/family (6 items). The spouse scale is divided into two dimensions: health factors (8 items) and social functioning factors (4 items). Using a Likert 5-point scale, higher scores indicate a higher level of fear of disease progression in patients. The Cronbach's α of patient and spouse scales were 0.886, 0.834 in the original research and 0.935, 0.719 in this study.

### Data collection

All participants were from the oncology and gastrointestinal surgery wards of two hospitals in China. After providing professional training to the researchers, the researchers recruited CRC patient couples who met the inclusion criteria in the wards. To avoid potential response bias, patients and their spousal caregivers were separately and privately invited to participate in the survey in a quiet area of the nurses' station. The researchers explained the purpose and methods of the survey to each participant in detail. After obtaining informed consent, the researchers distributed the paper-based Chinese version of the questionnaire to the participants. Each respondent completed the questionnaire anonymously and independently. During the survey, the researchers were present to help participants understand any confusing survey items. It took participants approximately 15–20 minutes to complete all the questionnaires. After the questionnaires were completed, the researchers checked the completeness of the data on the spot and corrected any errors promptly. All participants were informed that the collected data would be confidential and used only for research purposes.

### Data analysis

Data analysis was performed by using Mplus version 8.3 and IBM SPSS Statistics version 26.0. Using Mplus 8.3 for LPA to identify latent subgroups of DC strategies in CRC patients and their spousal caregivers. Starting from Model 1, the number of profiles in the model was gradually increased. The best model was determined based on the following fit indices: Akaike Information Criterion (AIC), Bayesian Information Criterion (BIC), and adjusted BIC (aBIC), which show a decreasing trend. Smaller values indicate better model fit.^35^ The entropy value closer to 1 indicates more precise classification.^36^ When both the Lo-Mendell-Rubin likelihood ratio test (LMR) and the bootstrap likelihood ratio test (BLRT) are significant (*P* < 0.05), it indicates that a model with k profiles is better than a model with k-1 profiles. Additionally, the sample size of the smallest profile in the model should not be less than 5%.^37^ Statistical description, χ^2^ test or Fisher's exact probability method, Wilcoxon signed-rank test, Kruskal–Wallis H test, analysis of variance, and ordered logistic regression analysis were performed using SPSS 26.0, with a significance level of *α* = 0.05.

### Ethical considerations

This study was approved by the Ethics Committee of Anhui Medical University (IRB No. 222359). Study procedures followed the principles set out by the Declaration of Helsinki, and all participants provided written informed consent.

## Results

### Participant characteristics

A total of 268 pairs of CRC patients and their spousal caregivers were included in this study. Among the patients, 67.2% were male and 32.8% were female, with a mean age of 61.99 ± 10.90 years. The average age of the spousal caregivers was 61.28 ± 10.95 years. The mean score of DC for CRC patients was 117.86 ± 10.28, and the mean score of DC for their spousal caregivers was 117.74 ± 9.81. Both scores fell within the range of 111–145, indicating that DC among CRC patient couples was at a moderate level. Details regarding the socio-demographic and clinical characteristics of the participants are shown in Table 1.

**Table 1:** Characteristics of participants (*N* = 268).

Variables	Patients, *n* (%)	Spousal caregivers, *n* (%)
Age (Mean ± SD, years)	61.99 ± 10.90	61.28 ± 10.95
Sex		
Male	180 (67.2)	88 (32.8)
Female	88 (32.8)	180 (67.2)
Religion		
No	258 (96.3)	265 (98.9)
Yes	10 (3.7)	3 (1.1)
Duration of marriage (Mean ± SD, years)		38.67 ± 11.60
Number of children		
0		1 (0.4)
1		67 (25.0)
≥ 2		200 (74.6)
Place of residence		
Countryside		103 (38.4)
Town		78 (29.1)
City		87 (32.5)
Education		
Primary school and below	118 (44.0)	135 (50.4)
Junior middle school	70 (26.1)	72 (26.9)
High or vocational school	42 (15.7)	37 (13.8)
College and above	38 (14.2)	24 (9.0)
Employment status		
Employed	46 (17.2)	50 (18.7)
Farmer	91 (34.0)	97 (36.2)
Retirement	74 (27.6)	64 (23.9)
Other	8 (3.0)	8 (3.0)
Unemployed	49 (18.3)	49 (18.3)
Monthly income per capita (RMB)		
＜2000		119 (44.4)
2000–5000		103 (38.4)
＞5000		46 (17.2)
Medical insurance type		
Employee medical insurance	77 (28.7)	
Resident medical insurance	187 (69.8)	
Commercial medical insurance	1 (0.4)	
At own expense	3 (1.1)	
Diagnosis duration		
＜3 months	158 (59.0)	
3–6 months	44 (16.4)	
6–12 months	27 (10.1)	
1–3 years	25 (9.3)	
＞3 years	14 (5.2)	
Type of stoma		
No stoma	209 (78.0)	
Ileostomy	43 (16.0)	
Colostomy	16 (6.0)	
Cancer stage		
Stage I	15 (5.6)	
Stage II	46 (17.2)	
Stage III	184 (68.7)	
Stage IV	23 (8.6)	
Type of chemotherapy		
No	151 (56.3)	
Yes	117 (43.7)	
Number of chronic diseases		
0		142 (53.0)
1		89 (33.2)
＞1		37 (13.8)
Score of DC (Mean ± SD)	117.86 ± 10.28	117.74 ± 9.81
Score of CRCP (Mean ± SD)	15.90 ± 2.03	16.35 ± 2.34
Score of FCR (Mean ± SD)	31.57 ± 2.34	31.89 ± 2.33

DC, dyadic coping; CRCP, cancer-related communication problems; FCR, fear of cancer recurrence.

### Classification of latent profile

**Fig. 1:**
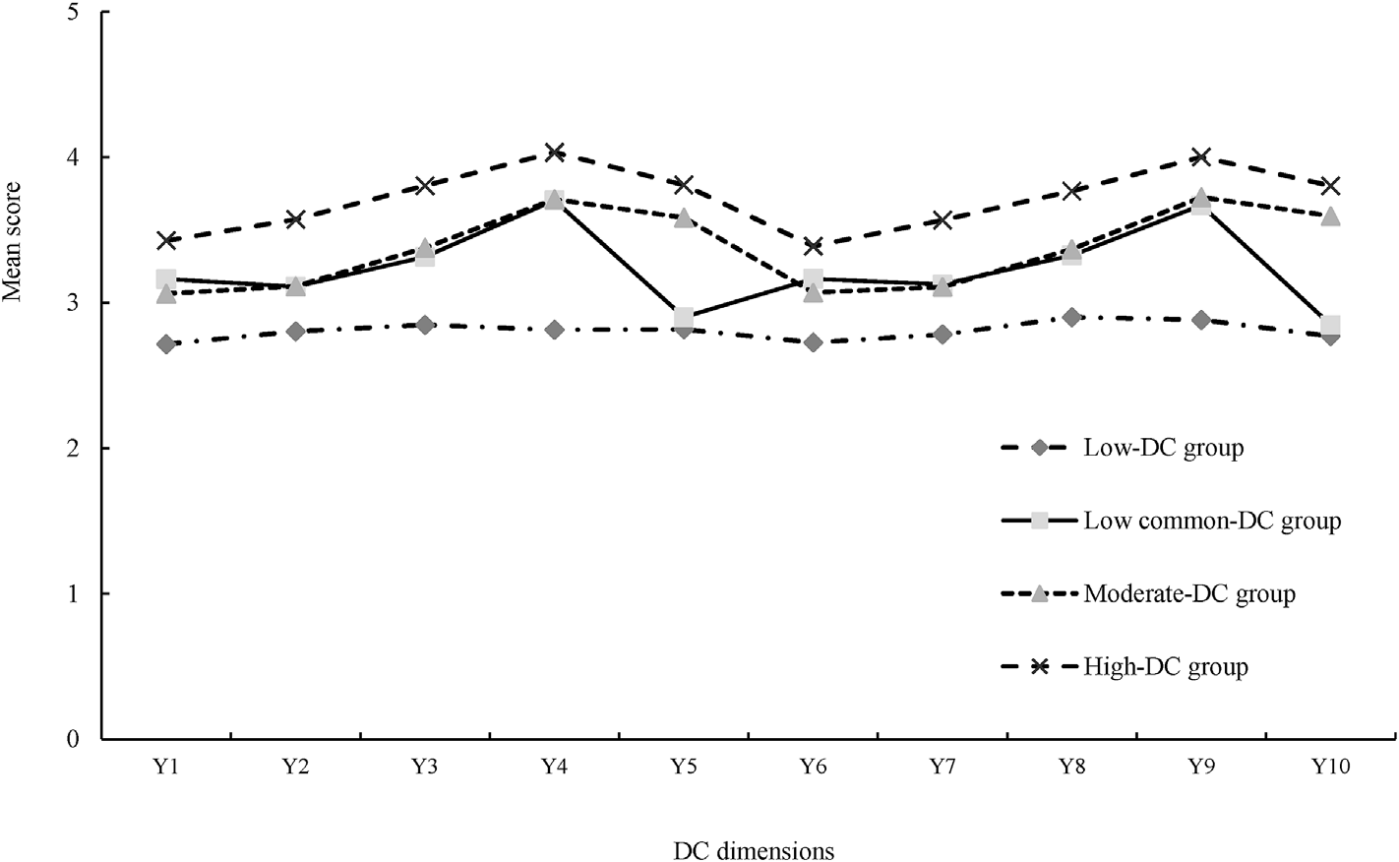
Four potential profiles of DC among CRC patients and their spouses. Y1, stress communication (patients); Y2, supportive coping (patients); Y3, delegated coping (patients); Y4, negative coping (patients); Y5, common coping (patients); Y6, stress communication (spousal caregivers); Y7, supportive coping (spousal caregivers); Y8, delegated coping (spousal caregivers); Y9, negative coping (spousal caregivers); Y10, common coping (spousal caregivers); DC, dyadic coping; CRC, colorectal cancer.

An LPA was conducted using the average scores of the dimensions of the DC scale for CRC patients and their spousal caregivers as manifest indicators, fitting a total of five latent profile models, as can be seen in Table 2. Starting from Model 1, the values of the fit indices AIC, BIC, and aBIC decreased with the increase in the number of profiles. When four profiles were reached, the Entropy value was closest to 1, and the LMR and BLRT values both showed *P* < 0.05, with the minimum class probability value not lower than 5%. Therefore, this study chose Model 4 as the best model. Based on this, a latent profile plot was drawn according to the mean scores of the items corresponding to the four categories (Fig. 1). The categories were named based on the score characteristics. Class 1 (C1) had low scores in all dimensions, reflecting a low level of DC in CRC patients and their spousal caregivers, hence it was named the “low-DC group”; Class 2 (C2) had low scores in common coping dimensions, reflecting low levels of common coping, hence it was named the “low common-DC group”; Class 3 (C3) had moderate scores in all dimensions, hence it was named the “moderate-DC group”; and Class 4 (C4) had relatively high scores in all dimensions, reflecting a high level of DC, hence it was named the “high-DC group”.

**Table 2:** Model fitting indexes of DC among CRC patients and their spousal caregivers.

Model	AIC	BIC	aBIC	Entropy	*P* (LMR)	*P* (BLRT)	Class probability (%)
1	11976.551	12048.37	11984.958	–	–	–	100.0
2	10559.694	10671.015	10572.726	0.995	＜0.001	＜0.001	18.7/81.3
3	9557.699	9708.521	9575.355	0.981	0.0016	＜0.001	18.3/53.0/28.7
4	9098.653	9288.975	9120.932	0.985	＜0.001	＜0.001	12.3/7.1/52.6/28.0
5	8930.056	9159.879	8956.959	0.976	0.0938	＜0.001	12.3/7.1/48.1/15.0/17.5

AIC, Akaike information criterion; BIC, Bayesian information criterion; aBIC, adjusted Bayesian information criterion; BLRT, bootstrapped likelihood ratio test; LMR, Lo-mendell-rubin; DC, dyadic coping; CRC, colorectal cancer.

As shown in Table 3, there were significant differences in the scores of DC dimensions between different subgroups (all *P* < 0.001), suggesting heterogeneity in DC among CRC patient couples in different subgroups.

**Table 3:** Differences in DC dimensions among the subgroups (Mean ± SD).

Variables	Total (*n* = 268)	Low-DC group (*n* = 33)	Low common-DC group (*n* = 19)	Moderate-DC group (*n* = 141)	High-DC group (*n* = 75)	*P*
Stress communication (patients)	3.13 ± 0.28	2.72 ± 0.10	3.16 ± 0.21	3.06 ± 0.20	3.43 ± 0.14	＜0.001
Supportive coping (patients)	3.20 ± 0.30	2.80 ± 0.12	3.11 ± 0.20	3.11 ± 0.17	3.57 ± 0.13	＜0.001
Delegated coping (patients)	3.43 ± 0.39	2.85 ± 0.21	3.32 ± 0.27	3.38 ± 0.27	3.81 ± 0.27	＜0.001
Negative coping (patients)	3.69 ± 0.42	2.81 ± 0.17	3.70 ± 0.17	3.71 ± 0.23	4.04 ± 0.24	＜0.001
Common coping (patients)	3.51 ± 0.37	2.82 ± 0.21	2.91 ± 0.22	3.59 ± 0.14	3.81 ± 0.09	＜0.001
DC (patients)	3.37 ± 0.29	2.79 ± 0.09	3.25 ± 0.12	3.34 ± 0.11	3.71 ± 0.12	＜0.001
Stress communication (spousal caregivers)	3.12 ± 0.26	2.73 ± 0.10	3.16 ± 0.24	3.07 ± 0.18	3.39 ± 0.13	＜0.001
Supportive coping (spousal caregivers)	3.20 ± 0.30	2.78 ± 0.10	3.13 ± 0.20	3.11 ± 0.17	3.57 ± 0.12	＜0.001
Delegated coping (spousal caregivers)	3.42 ± 0.36	2.90 ± 0.20	3.33 ± 0.22	3.37 ± 0.27	3.77 ± 0.22	＜0.001
Negative coping (spousal caregivers)	3.70 ± 0.39	2.88 ± 0.13	3.67 ± 0.23	3.73 ± 0.23	4.00 ± 0.21	＜0.001
Common coping (spousal caregivers)	3.50 ± 0.37	2.77 ± 0.09	2.85 ± 0.13	3.60 ± 0.09	3.81 ± 0.09	＜0.001
DC (spousal caregivers)	3.36 ± 0.28	2.80 ± 0.07	3.24 ± 0.12	3.34 ± 0.10	3.68 ± 0.09	＜0.001

DC, dyadic coping.

### Factors associated with symptom subgroups

Table 4 compared the demographic and clinical characteristics of the four subgroups. The results of the univariate analysis showed that age, education, medical insurance type, place of residence, monthly income per capita, number of children, number of chronic diseases in the spouse, type of stoma, cancer stage, couple illness communication, and FCR had statistical significance (*P* < 0.05).

**Table 4:** Differences in demographic and clinical characteristics among the subgroups.

Variables	Low-DC group (*n* = 33)	Low common-DC group (*n* = 19)	Moderate-DC group (*n* = 141)	High-DC group (*n* = 75)	*F*/ $\chi^{2}$/*H*	*P*
**Patients**						
Age (Mean ± SD, years)	56.91 ± 11.25^b^	61.32 ± 6.82	62.83 ± 11.34^a^	62.83 ± 10.29^a^	2.901	0.035
Gender					3.547	0.315
Male	18 (54.5)	14 (73.7)	94 (66.7)	54 (72.0)		
Female	15 (45.5)	5 (26.3)	47 (33.3)	21 (28.0)		
Religion					4.070	0.254
No	33 (100.0)	18 (94.7)	133 (94.3)	74 (98.7)		
Yes	0 (0.0)	1 (5.3)	8 (5.7)	1 (1.3)		
Education					29.937	＜0.001
Primary school and below	16 (48.5)^b^	12 (63.2)^b^	70 (49.6)^b^	20 (26.7)		
Junior middle school	10 (30.3)^b^	5 (26.3)^b^	41 (29.1)^b^	14 (18.7)		
High or vocational school	6 (18.2)^b^	1 (5.3)^b^	20 (14.2)^b^	15 (20.0)		
College and above	1 (3.0)^b^	1 (5.3)^b^	10 (7.1)^b^	26 (34.7)		
Employment status					1.950	0.583
Employed	4 (12.1)	2 (10.5)	22 (15.6)	18 (24.0)		
Farmer	12 (36.4)	9 (47.4)	56 (39.7)	14 (18.7)		
Retirement	5 (15.2)	5 (26.3)	28 (19.9)	36 (48.0)		
Other	2 (6.1)	1 (5.3)	3 (2.1)	2 (2.7)		
Unemployed	10 (30.3)	2 (10.5)	32 (22.7)	5 (6.7)		
Diagnosis duration					3.499	0.321
＜3 months	18 (54.5)	9 (47.4)	78 (55.3)	53 (70.7)		
3–6 months	7 (21.2)	4 (21.1)	28 (19.9)	5 (6.7)		
6–12 months	2 (6.1)	3 (15.8)	16 (11.3)	6 (8.0)		
1–3 years	5 (15.2)	3 (15.8)	12 (8.5)	5 (6.7)		
＞3 years	1 (3.0)	0 (0.0)	7 (5.0)	6 (8.0)		
Type of stoma					9.379	0.025
No stoma	32 (97.0)	14 (73.7)	102 (72.3)^a^	61 (81.3)		
Ileostomy	0 (0.0)	4 (21.1)	30 (21.3)^a^	9 (12.0)		
Colostomy	1 (3.0)	1 (5.3)	9 (6.4)^a^	5 (6.7)		
Medical insurance type					38.992	＜0.001
Employee medical insurance	2 (6.1)^b^	4 (21.1)^b^	30 (21.3)^b^	41 (54.7)		
Resident medical insurance	29 (87.9)^b^	15 (78.9)^b^	109 (77.3)^b^	34 (45.3)		
Commercial medical insurance	0 (0.0)^b^	0 (0.0)^b^	1 (0.7)^b^	0 (0.0)		
At own expense	2 (6.1)^b^	0 (0.0)^b^	1 (0.7)^b^	0 (0.0)		
Cancer stage					67.157	＜0.001
Stage I	0 (0.0)^b^	0 (0.0)^b^	3 (2.1)^a^^,^^b^	12 (16.0)		
Stage II	0 (0.0)^b^	1 (5.3)^b^	16 (11.3)^a^^,^^b^	29 (38.7)		
Stage III	21 (63.6)^b^	17 (89.5)^b^	115 (81.6)^a^^,^^b^	31 (41.3)		
Stage IV	12 (36.4)^b^	1 (5.3)^b^	7 (5.0)^a^^,^^b^	3 (4.0)		
Type of chemotherapy					5.606	0.132
No	15 (45.5)	89 (42.1)	79 (56.0)	49 (65.3)		
Yes	18 (54.5)	11 (57.9)	62 (44.0)	26 (34.7)		
Score of CRCP (Mean ± SD)	1.20 ± 0.09^b^	1.13 ± 0.10^b^	1.09 ± 0.10^a^^,^^b^	0.93 ± 0.12^a^	63.195	＜0.001
Score of FCR (Mean ± SD)	2.84 ± 0.12^b^	2.73 ± 0.17^b^	2.66 ± 0.17^a^^,^^b^	2.46 ± 0.12^a^	57.269	＜0.001
**Spouses**						
Age (Mean ± SD, years)	57.06 ± 11.18	61.16 ± 7.58	61.87 ± 11.14	62.04 ± 10.98	1.913	0.128
Religion					4.268	0.234
No	33 (100.0)	18 (94.7)	139 (98.6)	75 (100.0)		
Yes	0 (0.0)	1 (5.3)	2 (1.4)	0 (0.0)		
Education					41.079	＜0.001
Primary school and below	18 (54.5)^b^	14 (73.7)^b^	84 (59.6)^b^	19 (25.3)		
Junior middle school	13 (39.4)^b^	3 (15.8)^b^	36 (25.5)^b^	20 (26.7)		
High or vocational school	1 (3.0)^b^	2 (10.5)^b^	16 (11.3)^b^	18 (24.0)		
College and above	1 (3.0)^b^	0 (0.0)^b^	5 (3.5)^b^	18 (24.0)		
Employment status					2.806	0.423
Employed	7 (21.2)	2 (10.5)	25 (17.7)	16 (21.3)		
Farmer	15 (45.5)	7 (36.8)	61 (43.3)	14 (18.7)		
Retirement	2 (6.1)	3 (15.8)	25 (17.7)	34 (45.3)		
Other	1 (3.0)	1 (5.3)	4 (2.8)	2 (2.7)		
Unemployed	8 (24.2)	6 (31.6)	26 (18.4)	9 (12.0)		
Duration of marriage (Mean ± SD, years)	34.00 ± 11.89	38.89 ± 6.52	39.54 ± 11.87	39.04 ± 11.30	2.138	0.096
Number of children					16.377	0.001
0	0 (0.0)	0 (0.0)	0 (0.0)^b^	1 (1.3)		
1	12 (36.4)	5 (26.3)	22 (15.6)^b^	28 (37.3)		
≥2	21 (63.6)	14 (73.7)	119 (84.4)^b^	46 (61.3)		
Place of residence					26.951	＜0.001
Countryside	15 (45.5)^b^	10 (52.6)^b^	61 (43.3)^b^	17 (22.7)		
Town	13 (39.4)^b^	7 (36.8)^b^	44 (31.2)^b^	14 (18.7)		
City	5 (15.2)^b^	2 (10.5)^b^	36 (25.5)^b^	44 (58.7)		
Monthly income per capita (RMB)					49.253	＜0.001
＜2000	19 (57.6)^b^	12 (63.2)^b^	76 (53.9)^b^	12 (16.0)		
2000–5000	14 (42.4)^b^	6 (31.6)^b^	50 (35.5)^b^	33 (44.0)		
＞5000	0 (0.0)^b^	1 (5.3)^b^	15 (10.6)^b^	30 (40.0)		
Number of chronic diseases					18.285	＜0.001
0	20 (60.6)	8 (42.1)	62 (44.0)^b^	52 (69.3)		
1	10 (30.3)	9 (47.4)	49 (34.8)^b^	21 (28.0)		
＞1	3 (9.1)	2 (10.5)	30 (21.3)^b^	2 (2.7)		
Score of CRCP (Mean ± SD)	1.22 ± 0.08^b^	1.17 ± 0.10^b^	1.12 ± 0.14^a^^,^^b^	0.95 ± 0.11^a^	46.130	＜0.001
Score of FCR (Mean ± SD)	2.86 ± 0.12^b^	2.78 ± 0.18^b^	2.69 ± 0.17^a^^,^^b^	2.48 ± 0.12^a^	60.200	＜0.001

DC, dyadic coping; CRCP, cancer-related communication problems; FCR, fear of cancer recurrence.

a Compared to the Low-DC group, *P* < 0.05.

b Compared to the High-DC group, *P* < 0.05.

Taking the latent categories of DC in CRC patients and their spousal caregivers as the dependent variable (low-DC group = 1, low common-DC group = 2, moderate-DC group = 3, high-DC group = 4; with the high-DC group as the reference), logistic regression analysis was conducted using the variables with statistical significance from univariate analysis as independent variables. The test for parallel lines showed χ^2^ = 39.280, *P* = 0.120, indicating that ordinal logistic regression analysis could be used. The likelihood ratio test of the regression model showed χ^2^ = 210.193, *P* < 0.001, indicating that the model was effective. The results showed that disease stage (Stage I = 1, Stage II = 2, Stage III = 3, Stage IV = 4; with Stage IV as the reference), couple illness communication (raw scores input), and FCR (raw scores input) were influencing factors of the latent categories of CRC patients and their spousal caregivers, as shown in Table 5.

**Table 5:** Results of logistic regressions for the subgroups of DC.

Variables	B	SE	OR	95% CI	*P*
Low-DC group	−21.339	4.361	–	–	＜0.001
Low common-DC	−20.57	4.346	–	–	＜0.001
Moderate-DC group	−16.16	4.246	–	–	＜0.001
Cancer stage	−1.033	0.344	0.356	0.181–0.699	0.003
Couple illness communication	−7.374	2.375	0.001	0.000–0.066	0.002
FCR	−4.07	1.973	0.017	0.000–0.816	0.039

SE, standard error; OR, odds ratio; CI, confidence interval; DC, dyadic coping; FCR, fear of cancer recurrence.

## Discussion

### The DC of CRC patients and their spousal caregivers is at a moderate level

To our knowledge, this study is the first to explore the different subgroup patterns of DC in CRC patients and their spousal caregivers. The results indicate that the overall level of DC in CRC patients and their spousal caregivers are moderate, similar to the findings in other cancer patients,^38^ but lower than the DC scores of chronic disease patients.^39^ This may be due to the higher mortality rate of CRC compared to chronic diseases and the more severe public perception of the disease. Additionally, the long-term use of ostomy bags, the tortures of radiotherapy and chemotherapy, and the heavy caregiving and financial burdens impose significant psychological stress on both patients and their spousal caregivers, affecting their coping abilities.^40^^,^^41^ The stress communication dimension scored the lowest, influenced by the sensitivity of the disease and the implicit communication patterns in Chinese culture. CRC patients and their spousal caregivers may avoid discussing disease-related topics to prevent worrying their loved ones.^40^ Therefore, in clinical nursing, both patients and their spousal caregivers should be assessed, and dyadic nursing goals should be established. Encouraging emotional expression and mutual support between spouses can improve the level of DC in CRC patients and their spousal caregivers.

### There is heterogeneity in the DC of CRC patients and their spousal caregivers

This study classifies the DC of CRC patients and their spousal caregivers into four latent categories based on LPA: low-DC group, low common-DC group, moderate-DC group, and high-DC group, indicating heterogeneity in DC among CRC patients and their spousal caregivers.

The proportion of type C1 is 12.3%. Both CRC patients and their spouse caregivers scored low across various dimensions of DC, making them key targets for DC interventions. This category is characterized by low educational attainment, a monthly household income per capita of less than 2000 yuan, participation in residents' medical insurance, and cancer stages III-IV. The higher the cancer stage, the worse the prognosis. CRC patients and their spouse caregivers in this category, with relatively low incomes, are concerned not only about disease progression but also the high cost of medical treatment. Although China's medical security system is continuously improving, the reimbursement rate for residents' medical insurance at provincial tertiary hospitals remains relatively low. The heavy psychological and financial burden causes both parties to be reluctant to communicate, increasing their fear of the disease and resulting in lower DC levels, consistent with the findings of Ye et al.^42^ Further analysis revealed that, compared to types C3 and C4, lower patient age is more likely a characteristic of type C1. Younger patients may still be the main labor force in the family. Due to the disease, they are unable to work normally, worrying about prognosis and facing significant psychological and economic pressures,^43^ thus exhibiting lower DC with their spouse caregivers. Compared to type C3, type C1 has a higher proportion of patients without stomas. Stoma patients require more care from their spouses in stoma management, leading to more interaction in coping with cancer, but some study results are contrary to this finding,^44^ suggesting that the relationship between the two needs further verification.

The proportion of type C2 is 7.1%. Both patients and their spouse caregivers scored low on the dimension of common coping. This category is characterized by a high proportion of patients and spouse caregivers with an educational level of elementary school or below, predominantly living in rural areas, a monthly household income per capita of less than 2000 yuan, having two or more children, and a disease stage mostly at stage III. Patients and their spouse caregivers in this category have weaker abilities to actively or passively acquire disease-related knowledge and lower levels of intimacy in their relationships, ultimately affecting their common coping ability, consistent with the findings of Bai et al.^45^

Type C3 accounts for the largest proportion, 52.6%. Patients and their spouse caregivers scored at a moderate level across various dimensions of DC, indicating that the “moderate-DC group” may be the primary DC model for CRC patients and their spouse caregivers. This category is characterized by higher disease stages and shorter disease diagnosis durations. Further analysis revealed that, compared to type C1, type C3 is more likely to be associated with older patient age, the presence of stomas, a higher FCR, and fewer communication problems about the disease between spouses. Measures can be taken to improve communication between spouses and address the psychological FCR to further enhance DC levels.

Type C4 accounts for 28.0%. Patients and their spouse caregivers scored relatively high across various dimensions of DC. Compared to types C1, C2, and C3, this group is characterized by higher educational attainment (college degree or above), employee medical insurance, a monthly household income per capita of over 5000 yuan, and urban residency. Patients and their spouse caregivers in this category have a high level of disease awareness, strong self-management abilities, and access to more social resources during the disease treatment process, which reduces concerns about financial pressure. Couples with higher education levels feel more at ease in their intimate relationships, use more positive emotion regulation strategies, and maintain good communication and healthy psychological states while coping with the disease, resulting in stronger coping abilities, consistent with the findings of Mao et al.^46^ Further analysis revealed that having one child and a spouse caregiver without chronic diseases are more likely characteristics of type C4. The lower burden of having only one child and a spouse without chronic diseases may contribute to their stronger DC abilities.

### Influencing factors of DC in CRC patients and their spousal caregivers

#### The stage of the disease has a negative impact on the DC of CRC patients and their spousal caregivers

The stage of disease has a significant negative impact on CRC patients and their spousal caregivers. Compared to types C1, C2, and C3, type C4 patients have a lower tumor pathological grade and higher levels of DC with their spousal caregivers, consistent with the findings of Xu et al.^47^ Patients with higher tumor stages experience more severe symptoms, higher recurrence rates, and greater fear of the disease compared to early-stage patients, which increases the psychological burden on their spousal caregivers and weakens their coping abilities. Therefore, while managing patient symptoms, healthcare professionals should focus on reducing disease-related worry and fear for both the patient and their spousal caregivers. This can be achieved through sharing past treatment cases, cognitive-behavioral therapy, and supportive expressive therapy to enhance their confidence in disease prognosis, thereby improving the DC levels of both the patient and their spousal caregivers.

#### Couple illness communication has a positive impact on the DC of CRC patients and their spousal caregivers

Couple illness communication has a significantly positive impact on the DC of CRC patients and their spousal caregivers. Compared to types C1, C2, and C3, type C4 patients have better illness communication with their spousal caregivers, and higher levels of DC, consistent with the findings of Pan et al.^48^ Couple illness communication refers to the process in which the patient and their spouse exchange information related to the disease treatment and their personal feelings and concerns about the treatment.^49^ The emotional support expressed during the communication process can promote psychological adjustment following the diagnosis, effectively alleviate the immense stress caused by cancer, and increase both partners' ability to adapt to the illness, thereby improving their DC level.^19^ Therefore, healthcare providers should enhance the emotional expression, listening, and joint decision-making communication skills of patients and their spousal caregivers, fostering positive interactions and intimacy to jointly face the challenges of the disease.

#### FCR has a negative impact on the DC of CRC patients and their spousal caregivers

FCR has a significant negative impact on CRC patients and their spousal caregivers. Compared to types C1, C2, and C3, type C4 patients experience lower FCR with their spousal caregivers, and higher levels of DC, consistent with the findings of Soriano et al.^50^ FCR refers to the concern that cancer will recur in the same location or metastasize to other areas, leading to fear and worry about cancer recurrence.^51^ Studies have shown that FCR is also common among family caregivers, with spousal caregivers experiencing a higher level of this fear than cancer patients themselves.^52^ Persistently high levels of fear can exacerbate negative emotions in patients, leading to physical symptom disorders and thereby worsening the disease.^53^ It can also increase the psychological burden on spousal caregivers, affecting their caregiving abilities,^54^ which is detrimental to DC for both parties. Therefore, healthcare providers should offer targeted psychological counseling to both patients and spousal caregivers, helping them adopt positive coping strategies to quickly adapt to the traumatic event of cancer, thereby effectively improving DC levels for both.

### Implications for nursing practice and research

This study identified four subgroups of DC and influencing factors in CRC patients and their spousal caregivers. The findings had significant implications for enhancing the assessment and intervention of DC in CRC patients and their spousal caregivers. Influenced by Confucian culture, emotional expression between spouses in China is more reserved compared to Western countries, and couples are more prone to negative and pessimistic emotions when coping with illness. The study found that high disease stage, low educational level, and low monthly income per capita were characteristics of the low-DC group among couples. Nurses could use these characteristics to identify patients and spousal caregivers in the low-DC group, thereby better tailoring personalized support and interventions to help them cope with the negative impacts of cancer. Additionally, analyzing the influencing factors of DC in CRC patients and their spousal caregivers helped nurses identify potential health issues such as avoidant communication between couples and FCR, allowing for timely intervention and treatment. Nurse-led interventions in couple self-disclosure, cognitive behavioral therapy, and mindfulness-based stress reduction could effectively promote couple illness communication, alleviate FCR, and improve DC levels.

### Limitations

Nevertheless, this study has several limitations. Firstly, this study is a cross-sectional study, which cannot infer the potential causal relationships between variables or the dynamic changes in the different subgroups of DC among CRC patients and their spousal caregivers. Future longitudinal studies could explore the trends in DC changes and determine the optimal timing for interventions. Secondly, this study was conducted entirely in the form of questionnaires, with results self-reported by patients and their spousal caregivers, which is highly subjective and may lead to bias. Future research should combine subjective measurement results with objective measurement results to enhance the scientific validity of the findings. Lastly, the sample was sourced from a single region, which may limit the generalizability of the study results. Future research could conduct multi-center large-sample studies to make the findings more generalizable.

## Conclusions

The results of the study indicate that the DC of CRC patients and their spousal caregivers is at a moderate level. Using LPA, four subgroups of DC were identified: “low-DC group,” “low common-DC group,” “moderate-DC group,” and “high-DC group.” Healthcare professionals should focus on patients and spousal caregivers with advanced disease stages, insufficient couple illness communication, and severe FCR. Individualized interventions should be implemented based on the characteristics of different types to improve the DC levels of CRC patients and their spousal caregivers.

## CRediT authorship contribution statement

**Tingting Wei**: Conceptualization, Methodology, Data curation, Formal analysis, Writing. **Qiao Feng, Tingting A**: Methodology, Writing – Original draft preparation. **Ping Ni, Dongmei Zhuang**: Formal analysis, Writing – Revised draft preparation, Data curation. **Shaohua Hu, Shihui Yu**: Conceptualization, Methodology, Data collection, Writing – Original and Revised draft preparation. All authors had full access to all the data in the study, and the corresponding author had final responsibility for the decision to submit for publication. The corresponding author attests that all listed authors meet authorship criteria and that no others meeting the criteria have been omitted.

## Ethics statement

This study was approved by the Ethics Committee of Anhui Medical University (IRB No. 222359). All participants provided written informed consent.

## Funding

This study was supported by the Seedling Cultivation Project for Graduate Students of the School of Nursing, Anhui Medical University (Grant No. hlqm12024039), the Natural Science Research Project of Universities in Anhui Province (Grant No. KJ2021ZD0020), and the Provincial Quality Engineering Project of Higher Education Institutions in Anhui Province (Grant No. 2021jyxm0718). The funders had no role in considering the study design or in the collection, analysis, interpretation of data, writing of the report, or decision to submit the article for publication.

## Declaration of competing interest

The authors declare no conflict of interest.

## Data availability statement

The data that support the findings of this study are available from the corresponding author, Shaohua Hu, upon reasonable request.

## Declaration of generative AI and AI-assisted technologies in the writing process

No AI tools/services were used during the preparation of this work.
